# Plasmablastic Lymphoma with Coexistence of Chronic Lymphocytic Leukemia in an Immunocompetent Patient: A Case Report and Mini-Review

**DOI:** 10.1155/2017/2861596

**Published:** 2017-11-20

**Authors:** Eleftheria Hatzimichael, Konstantina Papathanasiou, Ioannis Zerdes, Stefanos Flindris, Alexandra Papoudou-Bai, Eleni Kapsali

**Affiliations:** ^1^Department of Hematology, University Hospital of Ioannina, Ioannina, Greece; ^2^Department of Pathology, University Hospital of Ioannina, Ioannina, Greece

## Abstract

**Background:**

Plasmablastic lymphoma (PBL) is a rare, aggressive B-cell lymphoma with poor prognosis usually found in the oral cavity of HIV-positive patients. Chronic lymphocytic leukemia (CLL) is an indolent B-cell lymphoma with a variable clinical course. Transformation of CLL to PBL as Richter's syndrome is rare while coexistence of CLL and PBL at diagnosis is even rarer.

**Case Report:**

We describe a case of a male immunocompetent patient with an ileum-cecum valve mass and a soft tissue mass at the left humerus with histologic evidence of PBL with coexistence of CLL in the bone marrow and peripheral blood. Amputation of the patient's left arm was inevitable, and the patient was started on bortezomib and dexamethasone. However, prolonged hospitalization was complicated by aspiration pneumonia, and the patient passed away.

**Conclusions:**

No standard of care exists for patients with PBL, and prognosis remains dismal. Concomitant presentation of hematological malignancies becomes increasingly recognized, and further insight is needed in order to delineate whether they originate from the same clone or from different ones.

## 1. Introduction

B-cell lymphomas with plasmablastic features are a heterogeneous group of lymphomas. While they share overlapping morphological or immunophenotypical features, distinct clinicopathological or molecular genetic features exist for some that have allowed their recognition as distinct entities [1]. Plasmablastic lymphoma (PBL) is an aggressive B-cell lymphoma that was relatively recently described. In the original report by Delecluse et al., most patients were HIV seropositive with a large B-cell lymphoma of the oral cavity with unique immunohistological features, mainly absence of the CD20 expression, constant expression of VS38c, and variable expression of CD79a [2]. The World Health Organization (WHO) classification specifically recognizes PBL as a distinct, aggressive non-Hodgkin B-cell neoplasm that shows diffuse proliferation of large neoplastic cells, resembling B immunoblasts with an immunophenotype of plasma cells [3, 4]. Over the last years, several case reports and small series have been published on both immunodeficient and immunocompetent patients involving various anatomic sites [5–9]. However, PBL remains a rare entity that has not been adequately described, a diagnostic challenge due to its similarities with multiple myeloma (MM), and a therapeutic challenge since no standard of care exists, and prognosis remains poor.

Chronic lymphocytic leukemia/small lymphocytic lymphoma (CLL/SLL) is an indolent B-cell lymphoma with a variable clinical course. CLL remains an incurable disease with relapses that become progressively more difficult to treat and with a shorter progression-free survival with each line of treatment. The reporting of other malignancies in patients with CLL is occurring in increasing frequency. Several retrospective studies have suggested that CLL patients carry a threefold risk of developing a secondary malignancy [10]. Commonly diagnosed secondary malignancies include Kaposi sarcoma and lung, breast, colon, and prostate cancer [11]. It is still not clear whether this increased risk is due to the underlying disease and accompanying chronic immunosuppression or due to the treatments given. Moreover, 5–10% of CLL patients may have their disease transformed to a more aggressive large cell lymphoma (Richter's transformation or Richter's syndrome, RS) [12, 13] or prolymphocytic leukemia [14], whereas transformation to PBL is extremely rare.

Coexistence of PBL with CLL has been described thus far in two patients [15, 16]. Holderness et al. described the first case of PBL arising in an HIV-negative, previously untreated CLL patient [15] who responded to brentuximab vedotin treatment as third-line treatment, while Ronchi et al. [16] most recently reported the simultaneous coexistence of PBL and CLL in the same lymph node in another HIV-negative, previously untreated CLL patient. Both patients had very poor overall survival (OS). To our knowledge, we report the third case of PBL arising in the setting of a previously untreated CLL in an immunocompetent and HIV-negative patient.

## 2. Case Report

A 67-year-old English male with an unremarkable past medical history (including any sexually transmitted diseases, HIV infection, or other immunosuppressive conditions) presented with a painful left arm. Physical examination revealed a palpable mass in his right abdomen and bilateral swelling of inguinal lymph nodes. An X-ray of the left upper limb revealed lytic bone lesions in the left humerus and a proximal humeral fracture surrounded by a mass. Computed tomography (CT) scan of the chest, abdomen, and pelvis showed left axillary, mesenteric and inguinal lymphadenopathy (maximum diameter 3.5 cm), along with a mass in the ileum-cecum valve. Full blood count revealed normocytic anemia (Hb 8.6 g/dl and MCV 81.1 fl) and white blood cell count of 16.3 × 10^9^/L with lymphocytic predominance (44%), whereas biochemical tests revealed hypercalcemia (10.8 mg/dl) and hypoalbuminemia (serum albumin 2.8 g/dl). Serum protein electrophoresis was normal and no Bence–Jones protein was detected in the urine.

**Figure 1:**
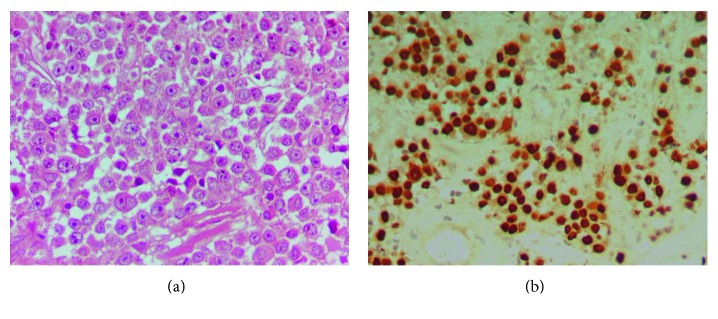
Plasmablastic lymphoma. The tumor cell was large with prominent nucleoli (a) (H&E staining, magnification ×600) and positive for Epstein–Barr virus by in situ hybridization with the EBER probe (b) (DAB, magnification ×400).

Biopsies from the polypoid mass of the ileum-cecum valve and the lytic lesion of the left humerus (Figure 1) revealed infiltration by neoplastic cells with plasmablastic traits and expression of CD38, CD138, MUM-1/IRF4, CD79a, and CD30. Other markers such as CD45, CD20, PAX5, CD56, CD5, CD23, ALK, LMP, EMA, cyclin D1, bcl6, and bcl2 were all negative. Kappa light restriction was observed. Ki-67 index was very high (75–85%). The tumor cells were positive for Epstein–Barr virus (EBV) by in situ hybridization (ISH) with the EBV-encoded small RNA (EBER) hybridization. The above findings along with the differential diagnosis of PBL by immunohistochemistry are shown in Table 1.

**Figure 2:**
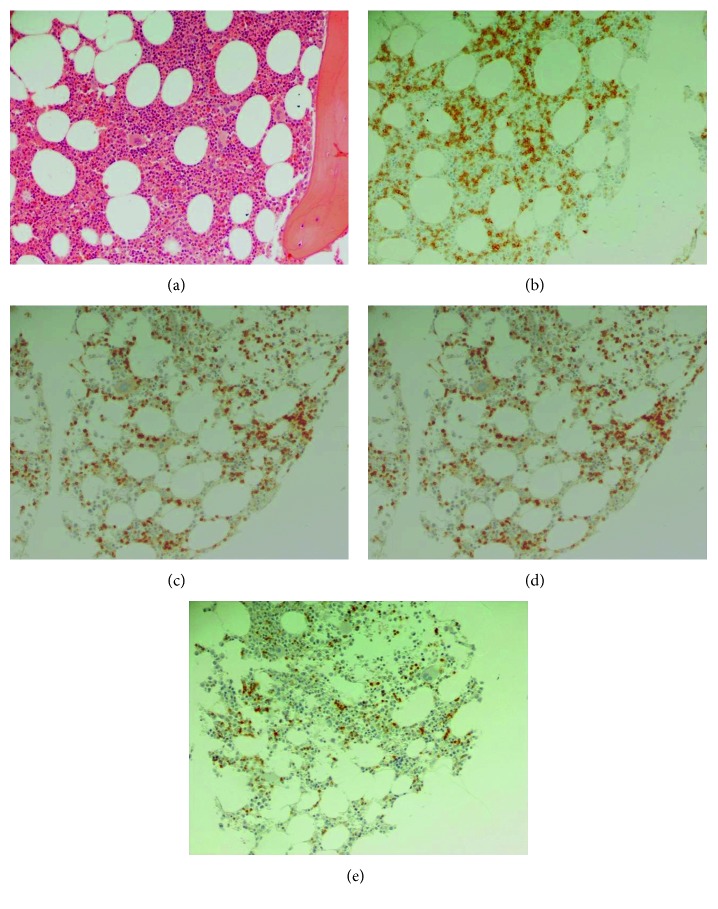
Bone marrow biopsy with limited interstitial infiltration by CLL. The neoplastic cells were small and round (a) (H&E staining, magnification ×200) expressing CD20 (b), CD5 (c), and CD23 (d) (DAB, magnification ×200).

Since an absolute lymphocytosis was noted, a flow cytometry was performed and was consistent with B-CLL (Matutes scoring system 4). In addition, bone marrow biopsy and its immunohistochemical analysis revealed limited (10–15%) interstitial infiltration of B cells (CD20+, CD23+, and CD5+) compatible with CLL (Figure 2). Bone marrow cytogenetics was normal (46, XY).

The patient was transferred to our institution, where further staging workup was performed. A second CT scan of the chest, abdomen, and pelvis revealed nodules (5 mm each) in the right and left lung and enlargement of mesenteric, retroperitoneal, inguinal, and paraortal lymph nodes. Excessive bilateral lytic lesions of the sacrum and of the left ilium pelvic bone were also observed.

Amputation of the patient's left arm was inevitable since the mass had infiltrated a large area of the humerus, and no reconstructive operation was feasible. Due to a gradually declining renal function, the patient was started on bortezomib (1.3 mg/m^2^ on days 1, 4, 8, and 11) and dexamethasone (40 mg on days 1–4, 8–11, and 15–18) with a plan to receive VCD (bortezomib, cyclophosphamide, and dexamethasone) for 4–6 cycles. The patient's poor performance status and worsening renal failure prohibited us from administering a more intensive chemotherapeutic regimen such as dose-adjusted EPOCH, hyper-CVAD, or CODOX-M/IVAC as suggested by the National Comprehensive Cancer Network. Unfortunately, chemotherapy and prolonged hospitalization were complicated by aspiration pneumonia, and the patient passed away 3 weeks after the initiation of treatment and 2.5 months post diagnosis.

## 3. Discussion

PBL is a rare entity of non-Hodgkin lymphoma (NHL) that was originally reported in the oral cavity and in the setting of HIV infection. However, it can also occur in other settings of immune compromise such as advanced age [17], post purine analogue therapy [18], or posttransplant [19]. Although prognosis in general is poor, varying between 3 and 12 months, administration of highly active antiretroviral therapy (HAART) in HIV seropositive patients has been shown to improve OS maybe due to the reconstitution of the immune response to the tumor [5, 19]. Although oral cavity has been reported as a major location of PBL, other extranodal sites have also been reported such as the small intestine and skin [20]. In a minority of patients, advanced clinical stage, B symptoms, and bone marrow involvement are present.

CLL/SLL is a low-grade B-cell lymphoma usually manifesting with an indolent, prolonged clinical course. However, in 3–8% of the cases, a transformation to a more aggressive lymphoma, known as RS, may be noted [13]. This transformation is most commonly of the diffuse large B-cell lymphoma (DLBCL) type, but transformation to classical Hodgkin lymphoma [21] or T-cell lymphoma has also been observed [22]. The transformed aggressive lymphoma may be clonally related to CLL or clonally unrelated, with the latter being associated with a longer median survival in a series of 86 patients [23]. However, whether clonally related or unrelated DLBCL occurring in the context of CLL has better treatment outcome should be determined in prospective studies.

Transformation of CLL/SLL to a PBL is rarely seen, and six cases have been reported thus far [18, 24–26]. Robak et al. reported on a patient with CLL who was previously treated with cladribine and experienced PBL transformation. It was shown that the two neoplasms were not clonally related but rather originated from different B-cell progenitors; therefore, the authors suggested that PBL represented an unusual variant of RS that arose as a second clone on the ground of severe immunosuppression following purine analogue treatment [18].

Martinez et al. [24] reported on three cases of HIV-, EBV-, and CMV-negative patients with CLL that transformed to PBL. Clonal relationship between the two tumors was suggested in 2 of the 3 patients since the same light chain expression was found and in one patient by immunoglobulin gene rearrangement studies. Two of the patients had received treatment for the CLL prior to the transformation [24].

A patient with relapsed refractory CLL who transformed to RS was treated with chimeric antigen receptor- (CAR-) modified T cells targeted for CD19 and later relapsed with a clonally related PBL [27], whereas most recently, Chan et al. reported on two cases with CLL who transformed to PBL following ibrutinib treatment [28]. Foo et al. [25] also described a CLL patient who received a purine analogue, fludarabine, and on relapse rituximab and subsequently developed a nasopharyngeal mass that was consistent with PBL and cervical adenopathy that was consistent with classical Hodgkin lymphoma, both EBV positive. Immunoglobulin gene rearrangement studies showed that tumors were not clonally related, and the authors suggested that these secondary lymphomas arising post chemotherapy should be considered as (iatrogenic) immunodeficiency-associated lymphoproliferative disorders and be separated from true RS.

Simultaneous presentation, that is, coexistence, of CLL and PBL at diagnosis in previously untreated and immunocompetent patients has been previously reported in two patients. Holderness et al. [15] presented an HIV-negative patient who was simultaneously diagnosed with PBL and CLL. Clonality was not studied. The patient was originally treated with R-CHOP to progressive disease, then with bortezomib, ifosfamide, etoposide, carboplatin, and radiotherapy again to progressive disease, and later was given one infusion of brentuximab vedotin to which he showed dramatic response, but did not receive any further treatment due to recurrent gastrointestinal bleeding. Ronchi et al. [16] described an unusual case of RS with the coexistence of PBL and SLL in the same lymph node at the time of the first diagnosis. Using needle cores from the two different areas of the lymph node and by employing immunoglobulin gene rearrangement studies, the authors showed that the two neoplasms were clonally related. The patient was serologically negative for HIV, EBV, and HHV-8. He was treated intensively with hyper-CVAD, but persistent disease was detected by PET/CT scan.

To our knowledge, this is the third case of previously untreated CLL in a non-HIV-infected patient, with a concomitant development of PBL and poor survival. Differential diagnosis of PBL occasionally is difficult, especially for extraoral cases and HIV- and EBV-negative cases. The differential diagnosis includes mainly the plasmablastic multiple myeloma (PMM). The tumor cells' characteristic morphology and immunophenotypic findings (Table 1), along with the clinical features and EBV positivity, favored the diagnosis of PBL.

Regarding treatment, there is no standard of care for patients with PBL. CHOP seems to be an inadequate therapy for aggressive PBL, and there is a need for more intensive therapeutic regimens. On the other hand, intensive regimens such as CODOX-M/IVAC or EPOCH although effective do not seem to improve OS [29].

Recently, except for a high incidence of MYC translocations [30], several other genetic abnormalities that have been previously described in MM undergoing blastic transformation have also been reported in PBL [31], providing a link between MM and PBL and a rational for myeloma-orientated treatment. Bortezomib remains the backbone of frontline treatment in MM inducing quick responses, reversing renal failure, and overcoming the effect of poor prognosis cytogenetics [32].

Given the patient's poor performance status, and worsening of his renal function, a combination of bortezomib and dexamethasone therapy was initially administered to our patient with a plan to proceed with a triplet, but unfortunately, the patient passed away due to aspiration pneumonia.

In conclusion, we describe one case of PBL with double appearance in the humerus and the ileum-cecum valve with concurrent CLL in an HIV negative, immunocompetent patient. Concomitant presentation of hematological malignancies becomes increasingly recognized, and further insight is needed in order to delineate whether they originate from the same clone or from different ones. Better understanding of the underlying biology and the clinicopathological characteristics will help us define prognosis and design better therapeutic strategies. Further studies are also needed in order to determine whether PBLs should be treated as plasma cell neoplasias or NHLs.

## Figures and Tables

**Table 1:** Differential diagnosis of PBL by immunohistochemistry.

Markers	Case presented	PBL	PMM
CD138	+	+	+
MUM-1/IRF4	+	+	+
CD79a	+	+/−	+/−
CD45	−	−/+	−
CD20	−	−/+	−/+
PAX5	−	−/+	−
CD56	−	−	+/−
EBV	+	+/−	−

PBL, plasmablastic lymphoma; PMM, plasmablastic multiple myeloma.
